# Machine Perfusion of Extended Criteria Donor Organs: Immunological Aspects

**DOI:** 10.3389/fimmu.2020.00192

**Published:** 2020-02-27

**Authors:** Mindaugas Kvietkauskas, Bettina Leber, Kestutis Strupas, Philipp Stiegler, Peter Schemmer

**Affiliations:** ^1^Department of General, Visceral and Transplant Surgery, Medical University of Graz, Graz, Austria; ^2^Faculty of Medicine, Vilnius University, Vilnius, Lithuania

**Keywords:** extended criteria donors, immunological rejection, machine perfusion, marginal organs, transplantation

## Abstract

Due to higher vulnerability and immunogenicity of extended criteria donor (ECD) organs used for organ transplantation (Tx), the discovery of new treatment strategies, involving tissue allorecognition pathways, is important. The implementation of machine perfusion (MP) led to improved estimation of the organ quality and introduced the possibility to achieve graft reconditioning prior to Tx. A significant number of experimental and clinical trials demonstrated increasing support for MP as a promising method of ECD organ preservation compared to classical static cold storage. MP reduced ischemia–reperfusion injury resulting in the protection from inadequate activation of innate immunity. However, there are no general agreements on MP protocols, and clinical application is limited. The objective of this comprehensive review is to summarize literature on immunological effects of MP of ECD organs based on experimental studies and clinical trials.

## Introduction

**Figure 1:**
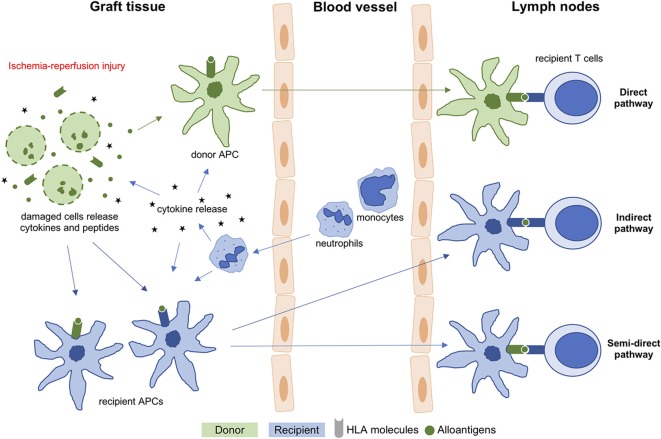
T cell allorecognition pathways in organ transplantation. APC, antigen-presenting cell; HLA, human leukocyte antigen.

The remarkable evolution of solid organ transplantation (Tx) has led to improved overall outcomes for patients with terminal organ dysfunction. However, ischemia–reperfusion injury (IRI) in combination with early immune activation remains a significant challenge limiting the potential of this therapy (1, 2). IRI depends on several factors, including primary condition of the graft and length of cold and warm ischemia time (CIT and WIT). It additionally determines the extent of the inflammatory response and increases immunogenicity and the degree of microcirculatory perfusion failure during reperfusion resulting in early allograft dysfunction or primary non-function (3, 4). As a link between the degree of IRI and activation of innate immunity (5) has been proposed, the discovery of new treatment strategies including tissue allorecognition pathways (Figure 1) has gained importance, especially in the era of extended criteria donor (ECD) organ Tx. The direct pathway starts with recipient CD4 and CD8 T cells recognizing endogenous alloantigens presented by donor human leukocyte antigen (HLA) molecules on the surface of donor antigen-presenting cells (APCs) after their migration from the graft to the recipient's lymph nodes. This process is initiated by the massive release of pro-inflammatory cytokines from damaged cells during IRI (4). On the other hand, the indirect allorecognition relies on recipient-derived APCs, which ingest, process, and present alloantigens (typically HLA antigens) in the context of recipient HLA, for self-restricted recognition by recipient T cells (6, 7). In the semi-direct pathway, recipient APCs acquire donor HLA molecules that present alloantigens directly to recipient T cells (8). Direct allorecognition alone can result in acute rejection, even without indirect mechanisms. Furthermore, depletion of donor immune cells from an organ prior to Tx may prevent rejection (9).

For more than 50 years, static cold storage (SCS) was the gold standard method for organ preservation until the interest in the concept of organ machine perfusion (MP) was renewed (10). To date, a significant number of experimental and clinical trials were published demonstrating increasing support of MP as a more physiologic method of solid organ preservation compared to SCS (11–15).

MP is a promising tool to reduce the gap between organ demand and supply that is resulting in a dramatic prolongation in waiting times and associated with increased morbidity and mortality for patients on the waiting list for Tx (16). In an effort to counter this trend, organ allografts that would have previously been deemed unsuitable are nowadays more frequently used for Tx (12) including donation after circulatory death (DCD) and ECD (aged ≥ 60 years or aged 50–59 years with vascular comorbidities) organs (12, 17, 18). Older donor organs have higher immunogenicity, mediated by poorer monocyte clearance of damaged necrotic cells, and therefore recipients may require a more intense immunosuppression in the early period after Tx (19–22). Knowing about the ECD grafts' increased risk for poor function or failure (23–25), implementation of new storage techniques, such as MP, paved the way for better characterization of organ quality and the possibility for graft reconditioning before Tx to improve organ vulnerability and immunogenicity (10, 26). MP reduced IRI in experimental and clinical models of ECD organ Tx resulting in protection from inadequate activation of innate immunity (1, 27–36).

**Figure 2:**
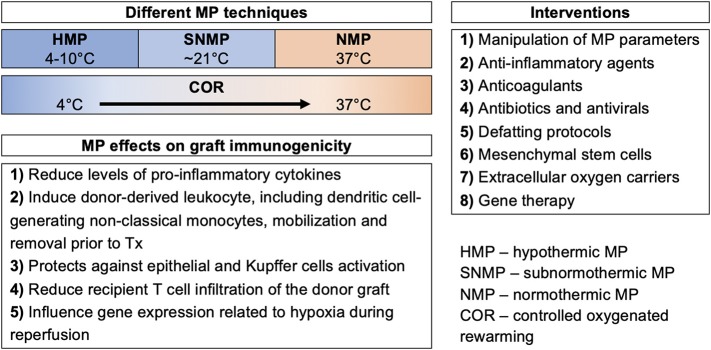
Different machine perfusion strategies of extended criteria donor organs for protection against activation of innate immunity.

Figure 2 summarizes frequently described MP settings including the underlying mechanisms. Briefly, hypothermic MP (HMP, 4–10°C) is based on the concept that oxidative energy production by mitochondrial electron transport is sustained at reduced rates by keeping low temperatures (10). In contrast, normothermic MP (NMP, 37°C) aims to provide an approximately near physiological environment for organs *ex vivo* (37). Subnormothermic MP (SNMP, ~21°C) is a halfway approach between HMP and NMP, while controlled oxygenated rewarming (COR) is a concept to rescue cold-stored marginal grafts by gentle oxygenated warming up prior to blood reperfusion (38, 39).

Currently, there are no general agreements on MP protocols, and clinical application is limited due to the lack of randomized clinical trials comparing the different MP strategies. The objective of this comprehensive review is to summarize literature on MP of ECD organs and discuss arising immunological aspects based on experimental studies and clinical trials.

## Machine Perfusion of Extended Criteria Donor Kidney Grafts

It seems that MP for Tx of ECD kidneys is associated with decreased IRI resulting in improved outcome compared to SCS (Table 1). Whereas most studies on MP in ECD kidneys reported positive effects on the graft, only a few studies reported inconclusive results (40, 48).

**Table 1:** Experimental and clinical studies of machine perfusion of extended criteria donor kidney grafts.

**Studies**	**Model**	**Primary graft condition, *N***	**MP time**	**Results and immunological aspects**
**ANIMAL STUDIES**				
Treckmann et al. (40)	Porcine HMP vs. retrograde oxygen persufflation vs. SCS with autoTx	DCD; *N* = 7/group WIT: 1 h	4 h	Malondialdehyde was dramatically increased in the MP kidneys on day 7, whereas levels in the other two groups were near normal values. The MP kidneys exhibited the most striking histological changes
Vaziri et al. (27)	Porcine HMP with Viaspan UW vs. KPS-1 vs. SCS without Tx	DCD; *N* = 7/group WIT: 1 h	24 h	HMP demonstrated superiority over SCS independently of perfusion solution. Results suggested significant benefits on graft outcome, particularly evident on the chronic effects of IRI with a protection against chronic immune response, epithelial to mesenchymal transition and interstitial fibrosis and tubular atrophy
Thuillier et al. (41)	Porcine HMP ± hyperoxia with Tx	DCD; *N* = 4/group WIT: 1 h	22 h	HMP with oxygen showed signs of higher quality and better function. Furthermore, the typical lesions of chronic graft loss were reduced, confirming improved ability to recover from the IRI
Stone et al. (1)	Porcine NMP without Tx	*N* = 10 CIT: 2 h	6 h	NMP initiated an inflammatory cytokine storm (especially IL-6, IFN-γ, and CXCL-8) and induced donor-derived leukocyte mobilization and removal prior to kidney Tx
Kasil et al. (42)	Porcine HMP ± M101 (2 g/L) ± hyperoxia with autoTx	DCD; *N* = 6/group WIT: 1 h	23 h	The M101 improved the HMP effect upon kidney recovery and late graft outcome. The infiltration of mast-cell leukocyte was nearly absent, leading to reduced fibrosis level in the kidney. Excess supply of oxygen has not improved the results
**HUMAN STUDIES**				
Reznik et al. (43)	HMP vs. SCS with Tx	Uncontrolled DCD; *N* = 17 vs. 21 WIT: 42.7 ± 1.6	12 h	A considerable number of complications and the negative effects, including acute rejection, correlated with the SCS group of kidneys
Treckmann et al. (44)	HMP vs. SCS with Tx	ECD; *N* = 91/group Median age: 66 y CIT: 13 h	n.d.	HMP preservation clearly reduced the risk of DGF and improved 1-year graft survival and function in ECD kidneys, while acute rejection rate was similar (17 vs. 16%, respectively)
Tozzi et al. (32)	HMP vs. SCS with Tx	Nyberg Score class C or D (donors mean age 67 ± 7 years); *N* = 10 vs. 13 CIT: 70 ± 25 min	12 ± 4 h	The levels of early inflammatory cytokines (TNF-α, IL-2, and IL-1β) were decreased in HMP group in perfusion and preservation liquid; however, there was a non-significant difference comparing sICAM-1
Nicholson et al. (45)	NMP vs. SCS with Tx	ECD; *N* = 10 vs. 47 CIT: ~11 h	63 ± 16 min	The incidence of acute rejection was similar in both groups (27.7 vs. 23.4%), while the delayed graft function rate was significantly reduced in the NMP group (5.6 vs. 36.2%)
Wszola et al. (46)	HMP vs. SCS	ECD vs. standard criteria donors; *N* = 62	24 h	MP influenced gene expression related to hypoxia during reperfusion and may improve the long-term results of kidney Tx
Wang et al. (47)	HMP vs. SCS with Tx	DCD and ECD; *N* = 24/group	5.86 ± 2.8 h	HMP reduced the incidence of DGF in DCD kidneys, and this effect is greater for ECD kidneys. Acute rejection rate was non-significantly different (4.1 vs. 8.3%, respectively)
Gallinat et al. (48)	End-ischemic HMP vs. SCS alone with Tx	ECD; *N* = 43/group Mean age: 66 vs. 67 years CIT: 13.4 vs. 12.1 years	1.6–12.8 h	PNF and DGF were 0 vs. 9.3% and 11.6 vs. 20.9%. There was no statistically significant difference in 1-year graft survival, while rejection rate within 3 months post Tx was significantly higher in the end-ischemic HMP group (38.5 vs. 10%, respectively)
Weissenbacher et al. (49)	NMP without Tx	DCD and DBD; *N* = 11 WIT: 16.2 ± 10 CIT: ~35 h	24 h	Demonstrated ability to maintain the condition of donor kidneys of ECD quality for long enough to carry out viability assessment and increase the feasibility to exploit this important source of donor organs
Ruiz-Hernández et al. (50)	Partial vs. total HMP with Tx	ECD; *N* = 119 vs. 74 Median age: 76.9 vs. 69.9 years CIT: 18.4 vs. 16.3 years	>4 h	There is a trend that complete HMP reduces the risk of DGF and improves 1-year graft survival in ECD kidneys
Savoye et al. (51)	HMP vs. SCS with Tx	ECD; *N* = 801 vs. 3,515 Mean age: 63.9 vs. 62.7 years CIT: 16.9 vs. 17.4 h	n.d.	Results confirmed the reduction in DGF occurrence among ECD kidneys preserved by HMP

*CIT, cold ischemia time; CXCL, C-X-C motif chemokine ligand; DGF, delayed graft function; ECD, extended criteria donor; DCD, donation after circulatory death; HMP, hypothermic MP; IFN, interferon; IL, interleukin; IRI, ischemia–reperfusion injury; MP, machine perfusion; NMP, normothermic MP; SCS, static cold storage; sICAM, soluble intracellular adhesion molecule; Tx, transplantation; WIT, warm ischemia time; UW, University of Wisconsin solution; PNF, primary graft nonfunction; DBD, donor after brain death*.

### Hypothermic Machine Perfusion Techniques

A DCD porcine kidney HMP model demonstrated improved graft outcome (27, 41, 42), particularly concerning the chronic effects of IRI by protecting against chronic immune response by reducing the epithelial to mesenchymal transition (27). Epithelial to mesenchymal transition plays an important role in the genesis of fibroblasts in the course of interstitial fibrosis (27, 52). Furthermore, oxygenated HMP showed superior outcome rates compared to non-oxygenated HMP (41). The significantly reduced occurrence of typical signs for chronic graft loss, like chronic inflammation or interstitial fibrosis, confirmed an improvement in recovery from IRI (41). Lately, the use of an extracellular oxygen transporter was investigated. M101 (hemoglobin of the marine worm) was associated with improved effects of HMP upon recovery and late graft outcome, shown by the nearly absent infiltration of mast cells resulting in reduced levels of fibrosis in the kidney (42). Extracellular oxygen carriers may logistically, rheologically, and immunologically be superior to packed red blood cells, but need further investigation. Studies on human DCD and ECD kidneys supported the superiority of HMP over SCS (32, 43, 45, 46). Reznik et al. (43) found a considerably lower number of complications and negative effects, like acute rejection, correlated with HMP kidneys retrieved from DCD donors. Another study in ECD kidneys (Nyberg Score class C or D) demonstrated an association of HMP with lower levels of early inflammatory cytokines [tumor necrosis factor (TNF)-α, interleukin (IL)-2, and IL-1β] in perfusion solution compared to SCS (32). HMP also affected the expression of hypoxia-related genes [i.e., hypoxia-inducible factor (HIF)-1α] (46). This may limit interstitial fibrosis and tubular atrophy, improving long-term outcomes in kidney Tx. ECD kidneys profited most by application of HMP (46).

### Normothermic Machine Perfusion Techniques

In a pig study, reduced graft immunogenicity was achieved by initiating an inflammatory cytokine storm [especially IL-6, interferon[108mmQ11 (IFN)-γ, and C-X-C motif chemokine ligand (CXCL)-8], leading to a donor-derived leukocyte mobilization and removal prior to kidney Tx (1). The authors proposed that migration of donor leukocytes in conjunction with the secretion of an IL-6, IFN-γ, and CXCL-8 storm leads to direct allorecognition and activates the recipient immune response following Tx (1). Short-term NMP of cold-stored human ECD kidneys did not reduce the incidence of acute rejection, while the rate of delayed graft function improved significantly (5.6 vs. 36.2%) (45). More recently, Weissenbacher et al. (49) was able to maintain the quality of ECD kidneys for up to 24 h, hence buying time for viability assessment, improving the feasibility to exploit this important source of donor organs using the NMP technique.

Although the primary results are encouraging, more research focusing on the reduction of immunogenicity of ECD organs is needed.

## Machine Perfusion of Extended Criteria Donor Liver Grafts

Currently, there is no general consensus on the standardized pretreatment of ECD livers in order to improve Tx outcomes (53). Experimental and clinical studies of MP of ECD livers are summarized in Table 2.

**Table 2:** Experimental and clinical studies of machine perfusion of extended criteria donor liver grafts.

**Studies**	**Model**	**Primary graft condition, *N***	**MP time**	**Results and immunological aspects**
**ANIMAL STUDIES**				
Lee et al. (54)	Rats HMP vs. SCS followed by 1 h machine reperfusion	DCD; *N* = n.d. WIT: 30 min	10 h	HMP for 10 h improved both function and microcirculation while reducing cellular damage of liver tissue when compared with SCS
Lauschke et al. (55)	Rats HMP with HTK vs. Belzer's solution vs. SCS followed by 45 min machine reperfusion	DCD; N ≥ 5/group WIT: 1 h	24 h	HLA class II antigen expression was detected on post-sinusoidal venular endothelium after SCS of DCD livers, while the antigen was almost absent or markedly reduced after HMP with HTK or Belzer's solution, respectively
Lee et al. (56)	Rats HMP vs. SCS with Tx	DCD; *N* = 7/group WIT: 30 min	5 h	HMP improved survival and reduced cellular damage of liver tissue that has experienced 30 min of WIT when compared with SCS tissues
Bessems et al. (57)	Rats HMP with Polysol or UW-G vs. SCS followed by 1 h machine reperfusion	DCD; *N* = 6/group WIT: 30 min	24 h	24 h HMP of DCD rat livers using the newly developed preservation solution Polysol results in less hepatocellular damage and better liver function compared to SCS in UW or HMP using UW-G
Manekeller et al. (58)	Rats HMP vs. SCS followed by 2 h machine reperfusion	DCD; N ≥ 5/group WIT: 30 min CIT: 16	0.5, 1, 2, and 3 h	1 h of post-conditioning after a long time (16 h) of SCS organs improved the viability and sustainability. The significantly higher ATP content and the lack of apoptotic signs in the tissue were observed
Nagrath et al. (59)	Rats NMP ± defatting agent cocktail without Tx	Steatotic livers, *N* = 7 vs. 5	3 h	Perfusate supplementation with defatting agents significantly reduced the intracellular fat content of perfused livers within a few hours
Olschewski et al. (60)	Rats HMP vs. SNMP vs. SCS without Tx	DCD; *N* = 5/group WIT: 1 h	6 h	In contrast to preservation at 4 or 12°C MP at 21°C has a beneficial positive effect on the initial organ function, structural integrity of the sinusoidal endothelium, and hepatocellular damage
Stegemann et al. (61, 62)	Rats HMP with different perfusion solutions vs. gaseous oxygen persufflation vs. SCS without Tx	DCD; *N* = 6/group WIT: 30 min	18 h	The use of Custodiol-N solution led to a significantly decreased release of ALT or LDH during HMP and reperfusion compared with HTK solution and reduced the level of apoptosis. The use of gaseous oxygen persufflation improved the tissue integrity and functional recovery of predamaged livers
Jamieson et al. (63)	Porcine NMP without Tx	Steatotic and normal livers, *N* = 3 vs. 5 WIT: 16 ± 4 min CIT: 76 ± 11 min	48 h	Steatotic livers can be successfully preserved using NMP for prolonged periods, and NMP facilitates a reduction in hepatic steatosis
Ferrigno et al. (30)	Rats SNMP vs. SCS followed by 2 h machine reperfusion	DCD; *N* = 5/group WIT: 30 min	6 h	MP preservation at 20°C improves cellular survival reducing the mitochondrial function in livers obtained from DCDs as compared with SCS
Gringeri et al. (31)	Porcine SNMP vs. SCS followed by 2 h machine reperfusion	DCD; *N* = 5/group WIT: 1 h	6 h	The SNMP group showed better histopathologic results with significantly less hepatic damage compared with SCS
Schlegel et al. (29)	Rats HOPE vs. SCS with Tx	DCD; *N* = 20/group WIT: 30 min CIT: 4 h	1 h	HOPE treatment significantly decreased IRI of hepatocytes by reducing the activation of Kupffer cells and endothelial cells. Moreover, HOPE-treated DCD livers were protected from activation of the innate immunity according to a decreased IRI
Schlegel et al. (64)	Porcine HMP with different parameters vs. SCS without Tx	DCD; *N* = 8/group WIT: 1 h CIT: 6 h	1 h	HOPE protected from mitochondrial and nuclear IRI by downregulation of the mitochondrial activity before reperfusion. Cold perfusion itself, under low-pressure conditions, prevented endothelial damage independently of oxygen
Izamis et al. (65)	Rats NMP with Tx	WIT: 0 vs. 1 h *N* = 11 vs. 7	5 h	MP suppressed lipid oxidation, likely due to the high insulin levels. Perfused livers did not consume all the available oxygen and were hypoxic independent of ischemic injury, suggesting that enhanced microcirculation via vasodilators and anti-thrombolytics might be an effective approach at optimizing the delivery of oxygen to hepatocytes
Minor et al. (38)	Porcine COR vs. HMP vs. SNMP vs. SCS	ECD; *N* = 6/group CIT: 18 h	1.5 h	COR significantly reduced cellular enzyme loss, gene expression and perfusate activities of TNF-α, radical mediated lipid peroxidation, and increase of portal vascular perfusion resistance upon reperfusion, while HMP or SNMP were less protective
Schlegel et al. (28)	Rats HOPE vs. deoxygenated MP with heterogenic Tx ± immunosuppression	CIT: 30 min	1 h	Study demonstrated that allograft treatment by HOPE not only protects against preservation injury but also impressively downregulates the immune system, blunting the alloimmune response
Bae et al. (33)	Rats HMP with KPS-1 vs. VAS ± VitE vs. SCS without Tx	DCD; *N* = 5/group WIT: 30 min	8 h	VAS perfusion solution was superior compared with KPS-1, and supplementation of VAS with VitE reduced not only the level of ALT but also levels of inflammatory cytokines (IL-6, TNF-α, and MCP-1) in graft tissue and caspase 3/7 in the circulation
Knaak et al. (39)	Porcine SNMP without Tx	DCD; *N* = 5 WIT: 45 min CIT: 4 h	6 h	SNMP minimized cold ischemic injury and allowed to assess ECD liver grafts prior to Tx
Nassar et al. (66)	Porcine NMP ± vasodilators (prostacyclin or adenosine) without Tx	DCD; *N* = 5/group WIT: 60 min	10 h	Livers perfused with the addition of prostacyclin showed a significantly higher outcome over those perfused by adding adenosine or without vasodilators, indicating the necessity of potent, efficient vasodilation in order to achieve effective preservation of DCD livers during NMP
Nassar et al. (67)	Porcine NMP vs. SNMP vs. SCS followed by 24 h machine reperfusion	DCD; *N* = 5/group WIT: 60 min	10 h	NMP was able to recover DCD livers showing superior hepatocellular integrity, biliary function, and microcirculation compared to SNMP and SCS
Ferrigno et al. (68)	Rats SNMP vs. SCS ± oxygenated washout Rats SNMP vs. SCS Both followed by 2 h machine reperfusion	DCD; *N* = 7/group WIT: 30 min Steatotic livers; *N* = 7/group	6 h	The use of oxygenated washout before SCS reversed liver injury in DCD organs, improving the ATP/ADP ratio; the use of MP did not otherwise prevent liver damage Using dynamic MP, a significantly lower hepatic damage and an increase in bile flow and in the ATP/ADP ratio were found compared with those of the SCS group
Chai et al. (69)	Rats HMP with UW ± metformin (0.165 mg/L) without Tx	Young and aged livers; *N* = 6/group	12 h	The addition of metformin to the UW preservation solution for *ex vivo* HMP reduced liver injury during cold ischemia, with significant protective effects on livers, especially of aged rats
Kron et al. (70)	Rats HOPE vs. SCS with Tx	Steatotic livers (≥60% macrosteatosis); *N* = 12/group CIT: 12 h	1 h	HOPE after cold storage of severely fatty livers significantly prevented reperfusion injury (less oxidative stress, nuclear injury, Kupffer and endothelial cell activation, as well as less fibrosis within 1 week after Tx) and improved graft function
Compagnon et al. (71)	Porcine HMP vs. SCS with Tx	DCD; *N* = 6/group WIT: 1 h	4 h	HMP-preserved livers functioned better and showed less hepatocellular and endothelial cell injury. In addition to improved energy metabolism, this protective effect was associated with an attenuation of inflammatory response, oxidative load, endoplasmic reticulum stress, mitochondrial damage, and apoptosis
Kakizaki et al. (72)	Porcine SNMP vs. SCS with Tx	DCD vs. DBD; *N* = 5/group WIT: 20 min CIT: 4 h	30 min	SNMP before Tx provided some recovery from IR injury in DCD liver grafts and significantly improved the survival rate
Nostedt et al. (73)	Porcine NMP after initial flush with different solutions and temperatures without Tx	DCD; *N* = 4/group WIT: 1 h	12 h	Avoiding initial hypothermia does not improve liver graft quality in a porcine DCD model of NMP
**HUMAN STUDIES**				
Henry et al. (34)	HMP vs. SCS with Tx	*N* = 18 vs. 15 WIT: 45.1 ± 6.3 min CIT: 9.3 ± 2.2 h	4.2 ± 0.9 h	HMP significantly reduced pro-inflammatory cytokine expression, relieving the downstream activation of adhesion molecules (ICAM-1) and migration of leukocytes, including neutrophils and macrophages, leading to improved overall outcomes
Bruinsma et al. (74)	SNMP without Tx	High-risk DCD and DBD; *N* = 7 WIT: ~28 min CIT: ~11.5 h	3 h	SNMP effectively maintained liver function with minimal injury and sustained or improved various hepatobiliary parameters post-ischemia
Dutkowski et al. (75)	HOPE vs. SCS with Tx	DCD; *N* = 50 vs. 25 WIT: ~35 min CIT: ~6.5 h	~2 h	HOPE protected extended DCD livers from initial reperfusion injury, leading to a better graft function and the prevention of intrahepatic biliary complications. Acute rejection rate was similar (16 vs. 12%)
Vogel et al. (76)	NMP without Tx	DCD (69%); *N* = 13 Mean age: 61.9 ± 11.3 years WIT: 11.3 ± 4 min CIT: 9.5 ± 3.7 h	24 h	They demonstrated the possibility to perfuse high-risk livers consistently for 24 h. The neutrophil infiltrate in grafts was eliminated after prolonged NMP
Laing et al. (77)	NMP with Hemopure* vs. RBC- based solution (matched) without Tx	High-risk (80% DCD); *N* = 5/group CIT: 7.5 h	6 h	Hemopure-based perfusion fluid is a feasible alternative to the blood-based solution currently used for liver NMP and may be logistically, rheologically, and immunologically superior to packed RBCs
Nasralla et al. (78)	NMP vs. SCS with Tx	DBD and DCD (~36%); *N* = 121 vs. 101	~9 h	NMP was associated with a 50% lower level of graft injury, measured by hepatocellular enzyme release, despite a 50% lower rate of organ discard and a 54% longer mean preservation time. There was no significant difference in bile duct complications, graft survival, or survival of the patient

*ALT, alanine aminotransaminase; CIT, cold ischemia time; COR, controlled oxygenated rewarming; ECD, extended criteria donor; DCD, donation after circulatory death; HLA, human leukocyte antigen; HMP, hypothermic MP; HOPE, hypothermic oxygenated perfusion; ICAM, intercellular adhesion molecule; IL, interleukin; IRI, ischemia–reperfusion injury; LDH, lactate dehydrogenase; MCP, monocyte chemoattractant protein; MP, machine perfusion; NMP, normothermic MP; RBC, red blood cell; SCS, static cold storage; SNMP, subnormothermic MP; TNF, tumor necrosis factor; Tx, transplantation; WIT, warm ischemia time; HTK, histidine-tryptophan-ketoglutarate solution; VAS, vasosol solution; DBD, donor after brain death*.

### Hypothermic Machine Perfusion Techniques

In several studies in DCD rat models, a reduction in IRI in liver tissue was evident after HMP when compared to SCS (28, 29, 54, 56, 57, 61, 62). This finding was confirmed in large domestic animal studies (64, 71). Hypothermic oxygenated perfusion (HOPE) treatment of DCD and severely fatty livers significantly decreased IRI of hepatocytes by reducing the activation of Kupffer and endothelial cells (29, 70). Moreover, HOPE successfully suppressed the recipient's immune system, blunting the alloimmune pathway (28, 29). This was evident by decreased Kupffer and endothelial cell activation induced by initial anti-oxidative effects and damage-associated molecular pattern (DAMP) release as a consequence of HOPE treatment and liver Tx (28). Furthermore, T cell infiltration in liver grafts as well as blood levels of circulating activated T cells decreased (28). A short time (1 h) of reconditioning of DCD rat and porcine livers using HMP after up to 16 h of SCS showed improvements in organ quality (58, 64). Long-term (24 h) HMP of DCD rat livers markedly reduced HLA class II antigen expression on post-sinusoidal venular endothelium compared to SCS (55). Bae et al. (33) found that supplementation of HMP perfusion solution with the antioxidant, vitamin E, reduced inflammatory cytokine levels [IL-6, TNF-α, and monocyte chemoattractant protein (MCP)-1], involved in alloimmune response, in graft tissue. The addition of metformin to HMP preservation solution reduced liver IRI, with significant protective effects on livers, especially in aged rats (69). Furthermore, HMP significantly reduced pro-inflammatory cytokine expression (TNF-α, IL-1β, and IL-8) (34). The attenuation of those cytokines affects many downstream pathways, including a reduced expression of chemokines and adhesion molecules such as intercellular adhesion molecule (ICAM)-1, MCP-1, P-selectin, and others. This effect subsequently decreases the level of neutrophil activation and inevitable leukocyte migration to stressed cell sites, leading to improved overall outcome rates in human livers (34). In another study, HOPE protected DCD livers from initial IRI, leading to improved graft function preventing intrahepatic biliary complications; however, acute rejection rate remained similar (16 vs. 12%) when compared to SCS (75).

### Subnormothermic/Normothermic Machine Perfusion Techniques

SNMP and NMP significantly ameliorated hepatic damage in DCD livers compared to SCS in animal models (31, 39, 60, 65, 68, 72). In a porcine model of liver MP, prolonged periods of NMP facilitate a reduction in hepatic steatosis (63), while the supplementation of perfusate with defatting agents significantly reduced the intracellular fat content of perfused rat livers within a few hours (59). Efficient vasodilation was found to be important in order to improve the effectiveness in the preservation of DCD livers during NMP (66). Olschewski et al. (60) compared HMP to SNMP and SCS, demonstrating beneficial effects on the initial organ function, structural integrity of the sinusoidal endothelium, and hepatocellular damage when DCD rat livers were perfused using SNMP. Furthermore, SNMP was associated with lower IRI when compared to SCS (74), while prolonged NMP additionally eliminated the neutrophil infiltrate in grafts (76). Another study of ECD livers showed superiority of COR over HMP, SNMP, and SCS (38). When comparing NMP to SNMP and SCS, NMP was most efficient in terms of recovery of DCD livers (67). Avoiding initial hypothermia did not improve liver graft quality in a porcine DCD model of NMP (73). Recently, the first randomized controlled trial showed a 50% reduction in liver graft injury, despite a 50% decrease in the number of discarded organs and a 54% increased mean preservation time after a period of NMP compared to SCS (~36% of grafts were DCD). However, they found no significant difference in bile duct complications, graft, or patient survival (78).

The currently ongoing VITTAL trial aims to improve the suitability of non-transplantable livers in the UK by monitoring their function during NMP followed by Tx of the sufficiently improved graft (79, 80). We expect that the results of this novel approach could improve consistency and increase the usage of ECD liver grafts without compromising recipient safety.

## Machine Perfusion of Extended Criteria Donor Lung Grafts

Experimental and clinical studies of ECD lungs and MP are compiled in Table 3.

**Table 3:** Experimental and clinical studies of machine perfusion of extended criteria donor lung grafts.

**Studies**	**Model**	**Primary graft condition, *N***	**MP time (h)**	**Results and immunological aspects**
**ANIMAL STUDIES**				
Nakajima et al. (81)	Canine HMP after SCS vs. SCS alone followed by 4 h machine reperfusion	DCD; *N* = 5/group WIT: 4 h CIT: 12 vs. 14 h	2	Short-term HMP could resuscitate ischemically damaged DCD lungs and ameliorate IRI. HMP significantly decreased oxidative damage and the production of pro-inflammatory cytokines after reperfusion compared with SCS
Mulloy et al. (82)	Porcine NMP vs. SCS vs. SCS + NMP with Tx. Perfusate supplemented with adenosine A2A receptor agonist	DCD; *N* = 5/group WIT: 60 min CIT: 4 h (SCS group)	4	The adenosine A2A receptor agonist exerts anti-inflammatory effects and reduces IRI when administered to DCD donor lungs during MP
Stone et al. (83)	Mice NMP ± A2A receptor agonist vs. SCS without Tx	DCD; *N* = 10–12/group WIT: 1 h CIT: 1 h	1	MP modulates pro-inflammatory genes and reduces pulmonary dysfunction, edema, pro-inflammatory cytokines, and neutrophil numbers in DCD lungs, which are further reduced by A2A receptor agonism
Stone et al. (9)	Porcine NMP vs. SCS with Tx	DCD; *N* = 12 WIT: 65 min CIT: 2 h	3	NMP resulted in reduction of donor leukocyte transfer into the recipient, and recipient T cell infiltration of the donor lung was significantly diminished
**HUMAN STUDIES**				
Stone et al. (36)	NMP without Tx	DCD; *N* = 7 WIT: 65 min CIT: 3 h	2	NMP showed the capacity to remove donor dendritic cell generating non-classical monocytes from graft
Nakajima et al. (35)	NMP ± broad-spectrum antibiotic without Tx	DBD with clinically diagnosed lung infection; *N* = 15 CIT: ~10 h	12	The results demonstrated that treatment with antibiotics significantly reduced bronchoalveolar lavage bacterial counts and inflammatory injury by decreasing endotoxin levels and key inflammatory mediators (TNF-α, IL-1β, MIP-1α, MIP-1β)
Nakajima et al. (84)	NMP ± MSCs with Tx	*N* = 6/group CIT: 24 h	12	The administration of MSCs ameliorated ischemic injury in donor lungs during NMP and attenuated the subsequent IRI after Tx

*CIT, cold ischemia time; ECD, extended criteria donor; DCD, donation after circulatory death; HMP, hypothermic MP; IL, interleukin; IRI, ischemia–reperfusion injury; MIP, macrophage inflammatory protein; MP, machine perfusion; MSCs, mesenchymal stromal cells; NMP, normothermic MP; SCS, static cold storage; TNF, tumor necrosis factor; Tx, transplantation; WIT, warm ischemia time; DBD, donor after brain death*.

### Hypothermic Machine Perfusion Techniques

Short-term HMP could resuscitate ischemically damaged DCD lungs and ameliorate IRI. In a canine model of MP, HMP improved the ATP production by the mitochondrial electron transport chain, leading to a significant decrease in oxidative damage and production of pro-inflammatory cytokines (IL-6 and TNF-α) after reperfusion compared to SCS (81). Moreover, short-term HMP washed out residual microthrombi in the donor lungs. All of those factors are important for Tx outcomes, including the reduction of the immunological rejection rate.

### Normothermic Machine Perfusion Techniques

NMP was able to modulate pro-inflammatory gene expression and reduce pulmonary dysfunction, edema, pro-inflammatory cytokines, and the number of neutrophils in animal DCD lungs (82, 83). Moreover, NMP resulted in reduced donor leukocyte transfer into the recipient by inducing mobilization of donor leukocytes into the perfusate and allowing their removal via the leukocyte filter prior to Tx (9). Therefore, reduced donor leukocyte migration to recipient lymph nodes resulted in a reduction of direct allorecognition and T cell priming, diminishing recipient T cell infiltration, the hallmark of acute rejection (9). In a clinical study, NMP showed the capacity to remove donor dendritic cells generating non-classical monocytes, which are directly involved in immune surveillance, from the graft (36). NMP of donor after brain death (DBD) lungs with clinically diagnosed infection significantly reduced bacterial counts in the fluid of the bronchoalveolar lavage and inflammatory injury by decreasing endotoxin levels and key inflammatory mediators [TNF-α, IL-1β, macrophage inflammatory protein (MIP)-1α, MIP-1β] when combined with broad-spectrum antibiotic treatment (35). The administration of mesenchymal stromal cells (MSCs) ameliorated ischemic injury in donor lungs during *ex vivo* NMP and attenuated the subsequent IRI after Tx (84).

The use of MP in reconditioning of ECD donor lungs for Tx is currently under investigation in clinical trials (85, 86), with results being expected soon.

## Machine Perfusion of Extended Criteria Donor Heart Grafts

Currently, clinical evidence of MP in ECD heart grafts is limited (Table 4). HMP improved the preservation of DCD heart grafts compared to SCS proven by superior post-reperfusion contractility. The underlying mechanisms could include enhanced preservation of the energetic states and superior cellular integrity (87). Recently, Korkmaz-Icöz et al. (88) demonstrated that HMP of aged donor hearts with MSCs protected against myocardial IRI in a rat model.

**Table 4:** Experimental and clinical studies of machine perfusion of extended criteria donor heart grafts.

**Studies**	**Model**	**Primary graft condition, *N***	**MP time (h)**	**Results and immunological aspects**
**ANIMAL STUDIES**				
Van Caenegem et al. (87)	Porcine HMP vs. SCS followed by 1 h machine reperfusion	DCD; *N* = 4/group WIT: 8–44 min	4	HMP improved the preservation of the heart grafts of DCD donors compared with SCS. This was proved by superior post-reperfusion contractility. The underlying mechanisms could include improved preservation of the energetic states and superior cellular integrity
Korkmaz-Icöz t al. (88)	Rats HMP ± MSCs with Tx	Aged donors; *N* = 6–9/group	5	HMP of donor hearts with MSCs protects against myocardial IRI in aged rats

*ECD, extended criteria donor; DCD, donation after circulatory death; HMP, hypothermic MP; IRI, ischemia–reperfusion injury; MP, machine perfusion; MSCs, mesenchymal stromal cells; SCS, static cold storage; Tx, transplantation; WIT, warm ischemia time*.

## Machine Perfusion of Extended Criteria Donor Pancreas Grafts

There is a limited number of studies evaluating the safety and feasibility of *ex situ* MP for ECD pancreas graft for whole-organ Tx (Table 5). HMP of porcine DCD pancreas was associated with a reduction in islet and acinar cell damage, stable perfusion dynamics, and minimal edematous weight change as well as potentially ameliorated endocrine viability and functionality after preservation (89, 90). More recent studies in the human pancreas indicated that especially DCD pancreas benefits more from oxygenated HMP compared to SCS alone (91). Even 24 h of HMP of ECD human pancreas–duodenum organs was feasible resulting in no deleterious parenchymal effects (92). Since those studies focused on the results after MP without following Tx, currently, there are no data available about clinical outcomes in this context.

**Table 5:** Experimental and clinical studies of machine perfusion of extended criteria donor pancreas grafts.

**Studies**	**Model**	**Primary graft condition, *N***	**MP time (h)**	**Results and immunological aspects**
**ANIMAL STUDIES**				
Karcz et al. (89)	Porcine HMP without Tx	DCD; *N* = 15 WIT: 25 min CIT: ~2.5 h	5:25	There was significant post-perfusion reduction in islet and acinar cell damage after HMP
Hamaoui et al. (90)	Porcine HMP after SCS vs. SCS alone followed by 2 h machine reperfusion	DCD; *N* = 3/group WIT: 30 min CIT: ~26.5 h	5	HMP-subjected grafts were associated with stable perfusion dynamics and minimal edematous weight change as well as potentially better endocrine viability and functionality
**HUMAN STUDIES**				
Leemkuil et al. (91)	HMP vs. SCS without Tx	Declined (DCD and DBD); *N* = 20 WIT: ~20 min CIT: ~4 h	6	This study indicated that especially the more injured DCD pancreas benefits more from oxygenated HMP compared with SCS alone
Branchereau et al. (92)	HMP vs. SCS without Tx	Rejected for organ or islet Tx; *N* = 7 vs. 2 WIT: n.d. CIT: n.d.	24	24 h of HMP of ECD human pancreas–duodenum organs was feasible with no deleterious parenchymal effect

*CIT, cold ischemia time; ECD, extended criteria donor; DCD, donation after circulatory death; HMP, hypothermic MP; MP, machine perfusion; SCS, static cold storage; Tx, transplantation; WIT, warm ischemia time; DBD, donor after brain death*.

## Conclusion

MP allows successful utilization of more vulnerable and immunogenic otherwise discarded ECD organs. It has been shown that MP not only reduces the levels of pro-inflammatory cytokines and positively influences gene expression related to hypoxia during reperfusion but also induces donor-derived leukocytes, including dendritic cell-generating non-classical monocytes, mobilization, and removal prior to Tx. Moreover, MP was able to protect against epithelial and Kupffer cell activation and to reduce recipient T cell infiltration of the donor graft. More recently, novel methods such as viral vector delivery during MP to allografts are under investigation (93). This biological modification of the graft prior to Tx may be a future therapeutic strategy to suppress the immune response against the allograft leading to Tx without or at least reduced dose of the systemic immunosuppression that carries the additional risk of infection and malignancy. Many studies have already shown superiority of ECD organ MP over the current standard SCS. However, there are no general agreements on MP protocols, and wider clinical application is limited due to the lack of randomized controlled trials. More trials focusing on immunological pathways in the different MP settings with respect to every single organ are mandatory to get detailed mechanistic insights. This knowledge about various pathways will help us to optimize organ quality after MP of ECD organs and therefore improve Tx outcomes as well as graft and patient survival.

## Author Contributions

All authors listed have made a substantial, direct and intellectual contribution to the work, and approved it for publication.

### Conflict of Interest

The authors declare that the research was conducted in the absence of any commercial or financial relationships that could be construed as a potential conflict of interest.
